# How to interpret the EURO-SKI study and its treatment-free remission outcome. Reply to R.P. Gale and J. Chen

**DOI:** 10.1038/s41375-023-02124-3

**Published:** 2024-01-03

**Authors:** Markus Pfirrmann, Susanne Saussele, François-Xavier Mahon, Johan Richter

**Affiliations:** 1 Institut für Medizinische Informationsverarbeitung, Biometrie und Epidemiologie (IBE), Medizinische Fakultät, Ludwig-Maximilians Universität München, Munich, Germany; 2 Department of Hematology and Oncology, University Hospital Mannheim, Heidelberg University, Mannheim, Germany; 3 Bergonié Cancer Institute, INSERM UMR1312 Inserm, University of Bordeaux, Bordeaux, France; 4 Department of Haematology, Oncology and Radiation Physics, Skåne University Hospital, Lund, Sweden

**Keywords:** Clinical trials, Chronic myeloid leukaemia, Epidemiology

## To the editor:

Recently, Gale and Chen published their perspective on “How should we interpret conclusions of TKI-stopping studies” [1]. The authors focused on the EURO-SKI trial as the largest study on treatment-free remission (TFR) after stopping a tyrosine kinase inhibitor (TKI), claimed the study to be biased, and criticised outcome interpretation. We explain why their allegation of bias is unjustified and clarify the interpretation of the trial.

## Study population

For the EURO-SKI trial starting May 2012, physicians from 61 centres in 11 European countries screened 868 patients with chronic myeloid leukaemia and included those who were eligible and consenting in the trial [2]. No existing “observational database” was used.

Gale and Chen entertained the suspicions that not all consecutively potentially eligible patients were screened and that subjects with the longest TKI-therapy or deep molecular response (DMR, BCR::ABL1 transcripts ≤ 0.01% on the International Scale, IS [3]) intervals were not included, knowing the trial involved stopping therapy [1]. These allegations lack any merit. Why should potentially eligible patients not have been screened by an investigator, who has agreed to the protocol of a trial on stopping therapy? And why precisely would inclusion depend on the length of treatment or DMR intervals? It was planned to register 500 patients within two years. In fact, 821 patients were registered within 2.5 years. Patients and physicians were more enthusiastic about the trial than hesitant for obscure reasons.

As a crucial measure against bias, the EURO-SKI trial was designed as a prospective trial. All values of baseline variables, including the candidate prognostic factors, were known at registration. In contrast, TFR outcome was observed post-registration. “Unavoidable limitations and biases” of observational studies do not apply to the EURO-SKI trial. At the time of inclusion, no subjective decisions regarding patient treatment were made. All patients uniformly discontinued TKI-treatment, irrespective of any (prognostic) patient profile. Physicians had no influence on the prospective outcome. The restart of TKI-therapy in 13 cases while still in major molecular response (MMR, BCR::ABL1 transcripts ≤ 0.1% on the IS) constituted the only deviations. Their influence on outcome was and is negligible (Fig. 3 in [2]).

## Interpretation of the results of the EURO-SKI trial

Gale and Chen questioned the representativeness of the EURO-SKI data, claiming that therapy outcomes in North America would fall behind those in Germany, referencing Radivoyevitch et al. [4]. However, these authors neither specifically addressed German patients nor conducted an adjusted comparison of molecular recurrence-free survival (MRecFS, recurrence: loss of MMR) between patient samples from different countries. Nevertheless, what really matters for future patients considering TKI-discontinuation is not the repeatability of MRecFS probabilities itself but rather the consistency of prognostic factors associated with MRecFS.

Indeed, the EURO-SKI patient sample is quite unique considering its size and its heterogeneity, with a median duration of TKI-therapy of 7.5 years (interquartile range [IQR]: 5.0–9.9 years) and a median duration of DMR before TKI-discontinuation of 4.7 years (IQR: 2.9–6.9 years). Sample size and heterogeneity made the EURO-SKI trial particularly suitable for studying prognostic factors. With 180 events (losses of MMR) in 405 patients, the sample size was clearly sufficient to examine 10 prognostic factors [5]. Sample size and the heterogeneity in the durations of TKI-therapy and DMR, enabled logistic regression based on a broad range of values, thus providing sufficient protection against bias in modelling. Even occasional deliberate protocol deviations in patient selection would not have introduced a substantial bias.

Considering the appropriateness of patients and temporal perspective for the EURO-SKI trial, survivorship bias is not relevant. The trial only addresses patients who were in DMR. The appropriate patients were basically defined by the inclusion and exclusion criteria (Table 1). Modelling MRecFS probabilities for all patients from the time of diagnosis is not reasonable since it included patients for whom discontinuation of therapy will never be relevant. It would also unnecessarily boost model uncertainty. The selection of prognostic factors for MRecFS probabilities should only depend on the patients the model is intended for.

**Table 1:** In- and exclusion criteria of the EURO-SKI trial [2].

BCR::ABL1-positive chronic-phase chronic myeloid leukaemia
Age 18 years or older
First-line TKI or second-line TKI because of toxicity with first-line TKI
Minimum treatment duration with a TKI at any dose of 3 years
Minimum duration of deep molecular response (DMR, BCR::ABL1 ≤ 0.01% on the International Scale [3]) of 1 year
At least 3 PCR results showing DMR within the year (give or take 2 months) before study entry and no loss of DMR during the same period
Typical BCR::ABL1 transcript (majority e13a2 or e14a2)
Known key variables: age, sex, date of diagnosis, start of first TKI, date of first DMR, dates of last 3 PCR results
No active concomitant malignancy
No previous allogeneic stem cell transplantation
No TKI failure according to the European LeukemiaNet recommendations of 2013 [7]
**Additional requirements for the EURO-SKI learning sample of 405 patients:**
First-line imatinib treatment
Knowledge of palpable spleen size enlargement in cm below the costal margin, platelet count, percentages of blasts, basophils, and eosinophils in peripheral blood. All values taken at diagnosis for prognostic score evaluation.
No interferon pre-treatment for > 1.5 years

*TKI* tyrosine kinase inhibitor.

The appropriate temporal perspective is the day when a patient in DMR discontinues TKI. On that very same day, say, patient A and patient B were in continuous DMR for 5 years and 8 years, respectively. In accordance with the model obtained in the EURO-SKI trial, their probabilities of staying in MMR 6 months after TKI-discontinuation are estimated to be 56 and 65% (Table 2). We cannot tell patient A her chances of MMR maintenance improve by 9% if she stays on TKI for 3 more years as she might lose DMR in between. Patients, who have maintained DMR for 8 years, are likely to constitute a prognostically favourable subgroup of all patients who had been in DMR for 5 years, 3 years ago. Still, since we do not know the risk of losing DMR within these 3 years, the chances of maintaining MMR might improve by postponing treatment discontinuation. Furthermore, keeping DMR while under TKI-treatment and keeping MMR without treatment are two different conditions. A direct transfer of the factors influencing response maintenance between the two different conditions is not possible.

**Table 2:** Proportion of patients maintaining MMR at 6 months after TKI-discontinuation in dependence on DMR duration before discontinuation.

Years in DMR before TKI-discontinuation	Estimated proportion maintaining MMR at 6 months (95% CI)
1	44% (35–53%)
2	47% (39–54%)
3	50% (44–56%)
4	53% (48–58%)
5	56% (51–61%)
6	59% (54–64%)
7	62% (55–68%)
8	65% (57–72%)
9	68% (58–76%)

*MMR* major molecular response, *BCR::ABL1* transcripts ≤ 0.1% on the International Scale [3], *TKI* tyrosine kinase inhibitor, *DMR* deep molecular response, *BCR::ABL1* transcripts ≤ 0.01% on the International Scale, *CI* confidence interval.

For 405 patients in the learning sample, the association between DMR duration before TKI-discontinuation and the probability of maintaining MMR at 6 months after TKI-discontinuation resulted in an odds ratio of 1.13 ([95% CI: 1.04–1.23]; *p* = 0·0032) [2]. By using the odds ratio in conjunction with the estimated intercept of –0.38 (95% CI: –0.82–0.06), it is possible to prognosticate the probability of maintaining MMR at 6 months from the logistic regression model. Of note, when estimating probabilities based on the logistic regression model averaging results across all 405 patients, 95% CIs are <20%. CIs > 30% result from the much smaller number of patients associated with specific intervals of DMR durations, as presented in Table 3 of Saussele et al. [2].

The EURO-SKI trial models the influence of different DMR durations in patients starting the second condition all on the same day. Like age, TKI and DMR duration have to be understood as fixed conditions when prognosticating the probability of MMR maintenance. Besides a somewhat elusive “prognostic profile”, there could be other unknown factors, not yet measured but potentially measurable in the future, hiding behind the duration of DMR.

## The bottom line

There is no indication of relevant bias in the EURO-SKI patient sample. With 62% MRecFS (95% confidence interval: 54–68%), the trial confirmed the feasibility of controlled TKI-discontinuation [2]. The significant association of DMR duration before TKI-discontinuation with MRecFS at 6 months was validated within the trial and, recently, in an independent Polish patient sample [6]. At least for patients meeting the criteria of Table 1, the results presented in Table 2 support the decision on treatment discontinuation based on DMR duration – whatever variables hide behind it.
